# Une loase oculaire: à propos d’un cas

**DOI:** 10.11604/pamj.2020.36.302.25124

**Published:** 2020-08-18

**Authors:** Nouhou Diori Adam, Yacoubou Soumana, Ali Saley, Idrissa Saley

**Affiliations:** 1 Service d’Ophtalmologie de l’Hôpital National Amirou Boubacar Diallo de Niamey, Niamey, Niger,; 2 Clinique Lumière Niamey, Niamey, Niger,; 3 Service d’Ophtalmologie de l’Hôpital Général de Référence Niamey, Niamey Niger,; 4 Service d’Ophtalmologie d’Omar Drissi Fès, Fès, Maroc

**Keywords:** Loase, conjonctivite, Niger, Loiasis, conjunctivitis, Niger

## Abstract

Notre objectif est de rapporter le cas d’une loase dans une zone sahélienne habituellement non endémique dans sa manifestation ophtalmologique. Il s’agissait d’un homme de 25 ans admis en consultation ophtalmologique pour sensation des corps étranger dans l’œil droit. Après examen ophtalmologique un ver translucide tortueux et mobile d’environ 4cm sous la conjonctive bulbaire à l’œil droit est observé. Après une extraction non traumatique chirurgicale, l’examen parasitologique confirme la loase. Il s’agit d’une parasitose des régions forestières essentiellement africaines. Suites aux mouvements des populations, elle peut être présente partout dans le monde. Il faut savoir la reconnaitre lors de nos consultations.

## Abstract (en)

The purpose of our study is to report a case of loiasis producing ocular manifestations in a patient living in the Sahel region, (non-endemic area). The study involved a 25-year old man admitted to ophthalmology consultation with foreign body sensation in the right eye. Ophthalmological examination objectified a tortuous, motile, translucent worm measuring around 4cm under the bulbar conjunctiva of the right eye. After surgical non-traumatic extraction, parasitological examination confirmed the diagnosis of loiasis, a parasitosis mainly occurring in African forests. As a result of population movements, it can occur everywhere in the world. It is essential to recognize it during consultations.

## Introduction

Plusieurs parasites ont un tropisme conjonctival avec soit des manifestations cliniques directes ou de réactions liées à leurs allergènes. La filaire *Loa loa*, parasite spécifiquement humain, est un ver rond, blanchâtre dont le male mesure 3.5cm et la femelle 5 à 7cm de long, vivant dans le tissu sous-cutané ou elle se déplace sans cesse par clivage des plans conjonctifs et musculaires [1]. La répartition géographique de la loase est strictement africaine où elle se limite à la zone équatoriale, au Cameroun et au Nigeria (régions forestières des pays d’Afrique Centrale de l’Ouest qui bordent le golfe de Guinée) [2]. Un interrogatoire minutieux est nécessaire sur la notion de séjour en zone d’endémie et un examen ophtalmologique complet pour orienter le diagnostic, guider et confirmé par un parasitologue. Nous rapportons un cas d’une *Loa loa* en zone sahélien dans sa manifestation sous conjonctivale chez un garçon de 25 ans au Niger.

## Patient et observation

**Figure 1:**
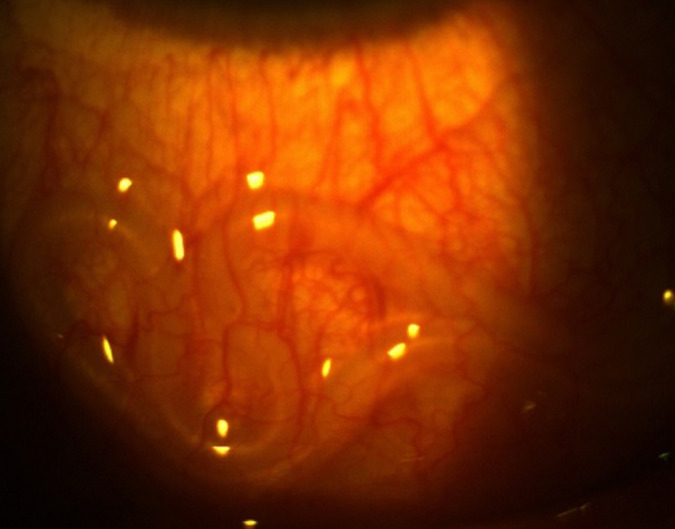
cordon blanc tortueux ondulant sous la conjonctive bulbaire

**Figure 2:**
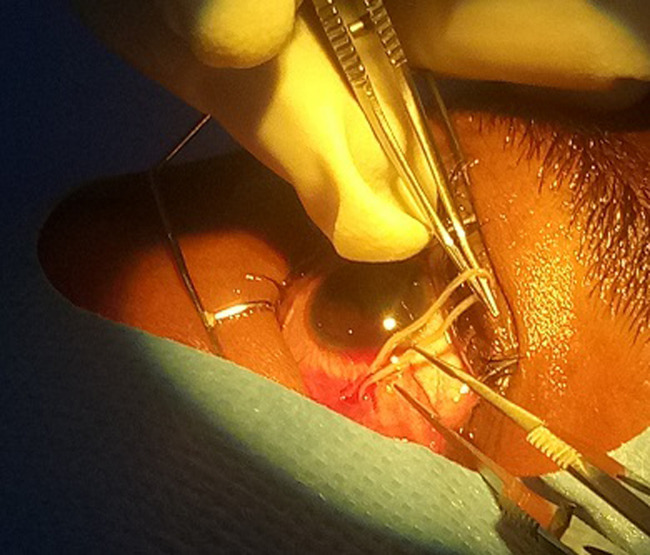
après désinsertion conjonctivale mise à nue du ver et saisie de la filaire à la pince Bonn pour diriger l’extraction

**Figure 3:**
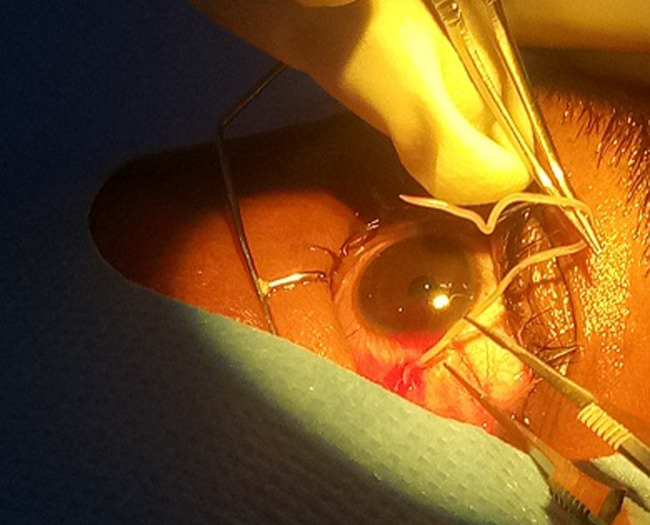
saisie de la filaire à la pince Bonn pour diriger l’extraction

**Figure 4:**
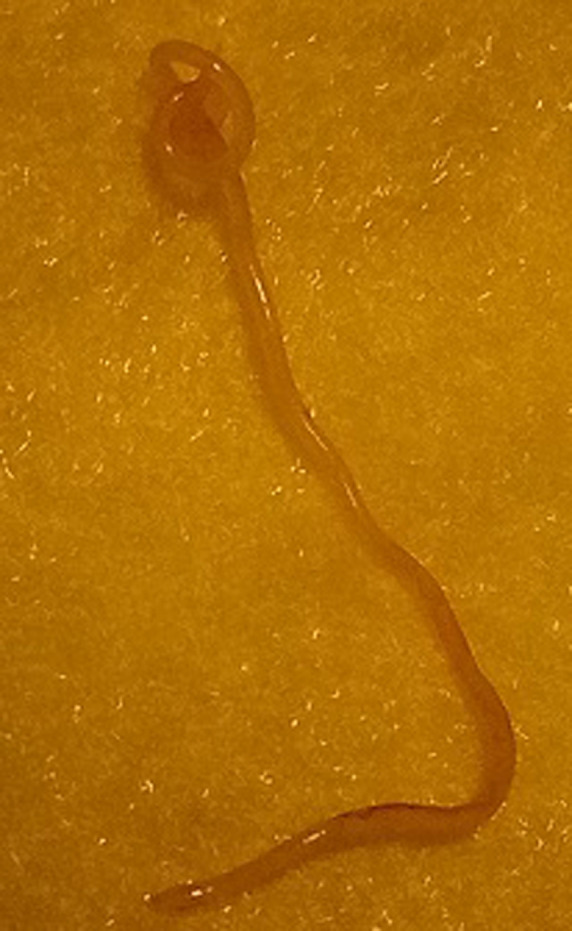
ver blanc tortueux après extraction

Il s’agit d’un homme âgé de 25 ans admis en consultation ophtalmologique pour sensation de corps étranger dans l’œil évoluant il y avait environs 3 jours selon le patient. On ne notait aucun antécédent ophtalmologique et autres connus. A l’examen on retrouvait une acuité visuelle conservée de 10/10 aux deux (2) yeux. A la lampe a fente on notait à l’œil droit une hyperhémie conjonctivale diffuse, on observait sous la conjonctive bulbaire un cordon blanc tortueux ondulant (Figure 1) et mobile. Le reste de l’examen était sans particularité. Devant ce tableau clinique une extraction chirurgicale non traumatique du ver était réalisée (Figure 2, Figure 3). Il s’agissait d’un ver vivant et d’environ 4cm de long translucide mobile (Figure 4) recueillie et adressé au laboratoire pour l’analyse où la *Loa loa* a été confirmée. Le patient a été mis systématiquement sous traitement par voie générale par Ivermectine® (21mg en une prise) puis par Albendazole, débuté 15 jours après (400mg deux fois par jour pendant 15 jours).

## Discussion

Le ver *Loa loa* est un nématode responsable d’une filariose bénigne de localisation sous-cutanée. Cette parasitose est endémique en Afrique centrale et de l’Ouest dans les forêts très humides tropicales [2]. Une fois dans le sang, la microfilaire peut être ingérée par un vecteur: mouche Chrysops [3]. Elle devient infectante en 10-12 jours [4]. Le vers adulte est responsable de la migration sous conjonctivale, sa taille est variable 3cm en moyenne, peut dépasser 10cm dans une étude en inde [5]. Les humains sont infectés lors de la piqure par la mouche. La microfilarémie est associée à des œdèmes Calabar [6]. Ceci a été retrouvé chez notre patient après un interrogatoire. Rarement les patients présentent une manifestation sous conjonctivales [7, 8]. Entrainant une réaction sous forme hyperhémie conjonctivales avec larmoiement, photophobie, sensation de corps étranger avec parfois perception par le patient du caractère mobile [1]. Comme a été le cas de notre patient. Exceptionnellement la filaire se retrouve dans la chambre antérieure. Les complications rénales, spléniques, cardiaques et neurologiques sont observées dans des formes à microfilarémie massives [9].

Le diagnostic positif repose dans un contexte clinique (séjour en zone endémique de *Loa loa*, et l’examen parasitologique du ver). Bien que le développement des voyages internationaux, à titre privé ou professionnel, implique de relativiser les notions de régions endémiques et non endémiques des maladies tropicales [8]. Son traitement est chirurgical dans les formes sous conjonctivales et cutané. Patient en décubitus dorsal, après application de la Bétadine, champage, mise en place d’un blepharostat, on procède à la désinfection des culs sacs conjonctivaux et bonne visualisation de la filaire. Après une instillation d’un anesthésique, création d’une boutonnière conjonctivo-tenonienne à l’aide d’une pince de Bonn et de ciseaux de castroviejo et en regard de la filaire. Désinsertion douce des tissus avec exposition partielle de la filaire. Saisie douce de la filaire afin de la libérer de ses attaches conjonctivo-tenonienne. Saisir la filaire à la pince Bonn pendant que l’on utilise les ciseaux de castroviejo pour diriger l’extraction. Enfin retirer le blepharostat et administration de collyre anti-inflammatoire et pommade puis pansement. Comme décrit par Mouinga Abayi DA *et al*. au Gabon en 2019 [10].

## Conclusion

L’avènement des migrations des populations n’épargne aucun pays du globe à la *Loa loa*. Sa description clinique oculaire bien que codifié et classique reste rare est spectaculaire. Tous praticien doit savoir la reconnaitre.

## References

[1] Pisella PJ, Assaraf E, Rossaza C, Limon S, Baudouin C, Richard-Lenoble D (1999). Conjonctive et parasitoses oculaires. J Fr Ophtalmol.

[2] Toufic N (1985). La loase et ses répercussions oculaires en Afrique centrales. Bull Soc Ophtalmol Fr.

[3] Orihel TC, Lowrie RC (1975). *Loa loa* development to the infective stage in an American deerfly, Chrysops atlanticus. Am J Trop Med Hyg.

[4] Flament J, Storck D (1997). Œil et pathologie générale: rapport de la société française d’ophtalmologie. Masson Paris.

[5] Mandal D, Roy D, Bera DK, Manna B (2013). Occurrence of gravid *Loa loa* in subconjunctival space of man: a case report from West Bengal. J Parasit Dis.

[6] Jain R, Chen JY, Butcher AR, Casson R, Selva D (2008). Subconjunctival *Loa loa* worm. Int J Infect Dis.

[7] Meda N, Dabouse MA, Djiguindé WP, Meda C, Konaté S (2009). Manifestations oculaires de la loase. J Fr d’ophtalmologie.

[8] Varenne F, Fillaux J, Porterie M, Soler J, Cassagne M, Soler V (2016). Loase sous conjonctivale: à propos d’un cas. J Fr ophtalmol.

[9] Ducorps M, Gardon-Wendel N, Ranque S, Ndong W, Boussinesq M, Gardon J (1995). Secondary effects of the treatment of hypermicrofilaremic loiasis using ivermectin. Bull Soc Pathol Exot.

[10] Mouinga Abayi DA, Mvé Mengome E (2019). Technique d’extraction d’une filasiose oculaire. J Fr Ophtalmol.

